# Obturator Neuropathy Following Minimally Invasive Pelvic Surgery: A Report of Two Cases

**DOI:** 10.7759/cureus.95220

**Published:** 2025-10-23

**Authors:** Collette Kokikian, Mimi Lam, Bhavesh Trikamji

**Affiliations:** 1 Neurology, University of California, Riverside School of Medicine, Riverside, USA; 2 Neurology/Neuromuscular Medicine, University of California, Los Angeles, Los Angeles, USA

**Keywords:** clinical neurophysiology, electromyography (emg), minimally invasive surgery, nerve conduction study (ncs), obturator nerve, pelvic surgery

## Abstract

Post-surgical neuropathies are uncommon but clinically significant complications of minimally invasive pelvic procedures. Obturator nerve injury, in particular, remains underrecognized in the context of hysterectomy and prostatectomy. We present two cases of iatrogenic obturator mononeuropathy and review the relevant literature. Case 1 involved a 45-year-old man with metastatic prostate adenocarcinoma who developed proximal left leg weakness one month after robotic-assisted radical prostatectomy. Case 2 involved a 40-year-old woman with grade 1 endometrial carcinoma who experienced similar right leg weakness following laparoscopic radical hysterectomy. Both patients reported weakness in the immediate postoperative period. Neurological examination demonstrated impaired thigh adduction with preserved hip and knee strength and intact reflexes. Imaging of the lumbar spine was unremarkable, while electromyography revealed obturator mononeuropathy. Rehabilitation resulted in complete functional recovery within six months. Obturator neuropathy is uncommon in patients following major pelvic surgery, most often due to intraoperative stretch, compression, or electrosurgical injury. Prompt recognition and rehabilitation are essential to optimize outcomes.

## Introduction

The obturator nerve arises from the lumbar plexus (L2-L4) and supplies motor innervation to the adductor muscles of the thigh and sensory innervation to the medial thigh [1]. Due to its anatomical course running posterior to the pelvic lymph node packet and along the lateral wall of the pelvis, it is vulnerable during dissection and manipulation in pelvic surgeries. Obturator nerve injury (ONI) is a rare yet significant complication associated with pelvic surgeries, particularly those involving pelvic lymph node dissection (PLND). Despite being reported in 0.2% to 5.7% of cases, ONI remains an under-recognized condition [1]. With the increasing use of minimally invasive techniques, such as robotic-assisted laparoscopic procedures, the incidence of ONI during surgeries such as radical prostatectomy and gynecologic oncologic interventions demands greater attention.

## Case presentation

Case 1

**Figure 1:**
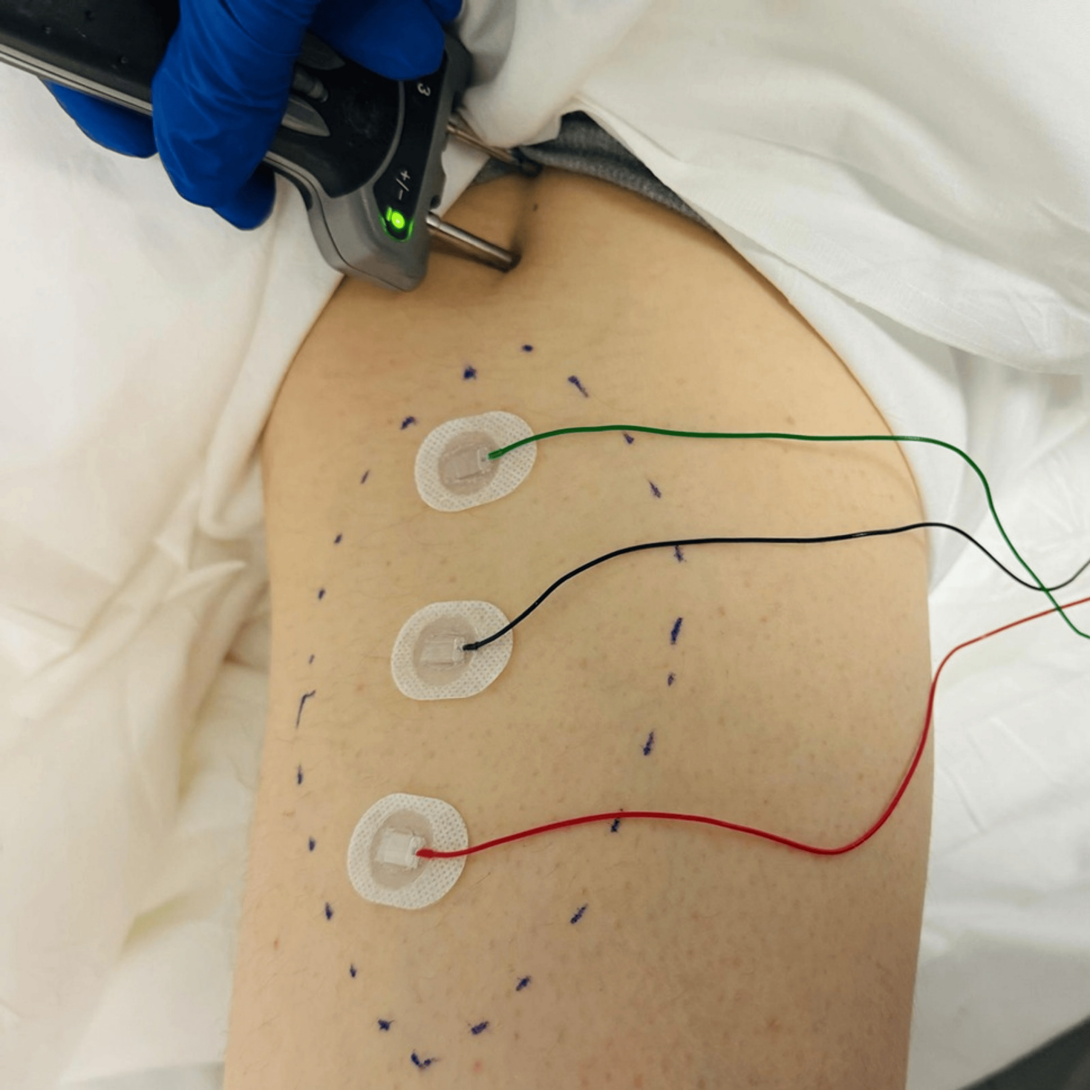
Obturator nerve conduction study for Case 1. Stimulation site: 1.5 cm inferior and 1.5 cm lateral to the pubic tubercle. Surface electrode placement at the midpoint of the medial thigh. Green cable: ground electrode; black cable: active electrode; red cable: reference electrode. Dotted line: area of sensory loss.

A 45-year-old male with newly diagnosed metastatic adenocarcinoma of the prostate (Gleason Grade 4 + 4 = 8) with involvement of all cores and the entire gland on MRI underwent an elective robotic-assisted laparoscopic radical prostatectomy and presented with left thigh weakness one month postoperatively. During the surgery, a bilateral extended node dissection was performed that included the external iliac, internal iliac, and obturator nodes to the level of the ureteral crossing. The nerve sparing was confirmed bilaterally during the procedure. Unfortunately, he reported weakness in left thigh adduction in the immediate postoperative period. This was associated with an area of numbness along the left medial thigh. MRI of the lumbar spine was found to be unremarkable. On neurological evaluation four weeks after the injury, he was noted to have 3/5 motor strength on Medical Research Council (MRC) grading on left thigh adduction with intact hip flexion, hip extension, knee flexion, and extension. There was an area of reduced pin sensation along the medial thigh. Reflexes were found to be normal and symmetric. Electrodiagnostic testing six weeks after the injury was notable for abnormal spontaneous activity and absent motor units in the left thigh adductors, confirming left obturator mononeuropathy (Figure 1, Tables 1, 2). The patient underwent structured rehabilitation with gradual resolution of symptoms six months after the injury.

**Table 1:** Motor nerve conduction study. MNC: motor nerve conduction; L: left; R: right; ms: milliseconds; mV: millivolts; m/s: meters per seconds; Med thigh: medial thigh; Add Mag: adductor magnus; NR: no response

MNC		Case 1		Case 2	
Nerve/Sites	Muscle	Latency (ms)	Amplitude (mV)	Latency (ms)	Amplitude (mV)
L. Obturator					
Med. thigh	Add Mag	3.60	0.1	2.91	2.1
R. Obturator					
Med. thigh	Add Mag	3.1	2.3	NR	NR

**Table 2:** Electromyography of Case 1. EMG: electromyography; L: left; IA: insertional activity; Fib: fibrillation potentials; PSW: positive sharp waves; Fasc: fasciculation potentials; MUAP: motor unit action potential; Amp: amplitude; Dur: duration; PPP: polyphasic potentials; N: normal

EMG summary table	Case 1							
	Spontaneous				MUAP			Recruitment
Muscle	IA	Fib	PSW	Fasc	Amp	Dur.	PPP	Pattern
L. Tibialis anterior	N	None	None	None	N	N	None	N
L. Gastrocnemius (medial head)	N	None	None	None	N	N	None	N
L. Peroneus longus	N	None	None	None	N	N	None	N
L. Tibialis posterior	N	None	None	None	N	N	None	N
L. Vastus medialis	N	None	None	None	N	N	None	N
L. Adductor magnus	2+	3+	2+	None	No units			
L. Biceps femoris (short head)	N	None	None	None	N	N	None	N
L. Biceps femoris (long head)	N	None	None	None	N	N	None	N
L. Gluteus medius	N	None	None	None	N	N	None	N
L. Gluteus maximus	N	None	None	None	N	N	None	N
L. L3 paraspinal	N	None	None	None	N	N	None	N
L. L5 paraspinal	N	None	None	None	N	N	None	N

Case 2

A 40-year-old female with grade 1 endometrial cancer underwent an elective laparoscopic radical hysterectomy under general anesthesia. She noticed weakness in right thigh adduction along with numbness in her medial thigh during recovery in the postoperative unit. Immediate MRI studies of her lumbar spine and right lower extremity were unrevealing for any central pathology. Upon neurological evaluation a month later, she was noted to have 3/5 strength on right thigh adduction on the MRC scale. The remainder of the neurological examination was normal. Electrodiagnostic studies two months after the injury revealed abnormal spontaneous activity and absent motor units in the right obturator innervated muscles, providing evidence for active right obturator neuropathy (Tables 1, 3). She underwent intensive rehabilitation, resulting in complete resolution of symptoms six months after the injury.

**Table 3:** Electromyography of Case 2. EMG: electromyography; R: right; IA: insertional activity; Fib: fibrillation potentials; PSW: positive sharp waves; Fasc: fasciculation potentials; MUAP: motor unit action potential; Amp: amplitude; Dur: duration; PPP: polyphasic potentials; N: normal

EMG summary table	Case 2							
	Spontaneous				MUAP			Recruitment
Muscle	IA	Fib	PSW	Fasc	Amp	Dur.	PPP	Pattern
R. Tibialis anterior	N	None	None	None	N	N	None	N
R. Gastrocnemius (medial head)	N	None	None	None	N	N	None	N
R. Peroneus longus	N	None	None	None	N	N	None	N
R. Tibialis posterior	N	None	None	None	N	N	None	N
R. Vastus medialis	N	None	None	None	N	N	None	N
R. Adductor magnus	3+	3+	3+	None	No units			
R. Biceps femoris (short head)	N	None	None	None	N	N	None	N
R. Biceps femoris (long head)	N	None	None	None	N	N	None	N
R. Gluteus medius	N	None	None	None	N	N	None	N
R. Gluteus maximus	N	None	None	None	N	N	None	N
R. L3 paraspinal	N	None	None	None	N	N	None	N
R. L5 paraspinal	N	None	None	None	N	N	None	N

## Discussion

Iatrogenic neuropathies affecting the lower extremities have been reported before under various circumstances [2]. ONI occurs most commonly during PLND, often as part of surgical treatment for gynecologic or urologic malignancies. In robotic-assisted laparoscopic prostatectomy, the incidence is approximately 0.4% [1]. It is also noted in gynecologic surgeries involving retroperitoneal dissection, endometriosis treatment, or paravaginal defect repair [3]. Some of the common risk factors include excessive medial traction during dissection, improper identification of anatomical landmarks, use of monopolar electrocautery, prolonged operative time, and inadequate patient positioning, especially during lithotomy [1,4-6]. ONI can result from several mechanisms, such as direct trauma (sharp or blunt dissection), stretch injury due to traction or limb positioning, thermal injury from energy devices, nerve ligation or crushing, and compression from hematomas or surgical hardware [4].

Prevention hinges on careful surgical planning and technique. Key strategies include identifying the obturator nerve before lymphadenectomy or deep pelvic dissection, using bipolar rather than monopolar cautery to minimize thermal injury, avoiding excessive traction during lymph node retrieval, and maintaining proper lithotomy positioning with at least 45° hip flexion when abduction exceeds 30° [1,4,7]. Cadaveric studies confirm that lower limb positioning can significantly strain the obturator nerve, making positioning a modifiable risk factor [7].

Diagnosis of ONI is primarily clinical, based on symptoms such as medial thigh pain, adduction weakness, and gait abnormalities. Clinical examination is supported by electrodiagnostic testing, such as electromyography, which routinely demonstrates denervation of obturator-innervated muscles (thigh adductors), but changes appear only after two to four weeks. Imaging modalities such as MRI and CT are useful when mass effect (tumor or hematoma) is suspected.

Management depends on the timing of diagnosis. Intraoperative recognition requires immediate microsurgical repair using fine nylon sutures (8-0 to 10-0) with precise epineurial alignment to avoid fascicular mismatch [3]. Postoperative recognition warrants initiation of a comprehensive physiotherapy program. This may include neuromuscular electrical stimulation, electromyographic biofeedback, muscle strengthening exercises, or a structured home treatment plan [3]. Studies show that early intraoperative repair has a high success rate, with most patients achieving full motor recovery within six months [1]. Even delayed diagnoses can yield good outcomes with appropriate rehabilitation [3].

ONI generally has a favorable prognosis with timely and appropriate intervention. A study involving 179 patients undergoing robot-assisted laparoscopic radical prostatectomy and radical cystectomy reported a 1.68% incidence of postoperative neuropathy, including ONIs, with all patients remaining ambulatory [8]. Another study showed that increased intraoperative time correlated significantly with neuropathy development, suggesting surgical efficiency as another modifiable factor [5]. Surgical series have also demonstrated improved symptoms postoperatively in patients undergoing obturator nerve decompression or repair, with resolution of pain and improved adductor strength [1,4,9].

Given its rarity and deep anatomical location, ONI may be overlooked unless clinicians maintain a high index of suspicion. Orthopedic, gynecologic, and urologic procedures, especially those involving endopelvic dissection, should involve routine nerve identification and preservation techniques [4]. Awareness of the obturator nerve’s location and risk factors is essential for surgeons and anesthesiologists to reduce the likelihood of injury.

## Conclusions

ONI is a rare but important complication of minimally invasive pelvic surgery. Despite technological advances, ONI remains a risk during PLND and other pelvic procedures. Surgical teams should employ meticulous technique, prioritize anatomical identification, and ensure proper patient positioning to reduce incidence. When injury occurs, early recognition and intervention, especially intraoperative repair, are key to ensuring optimal recovery. Electromyography and physiotherapy play crucial roles in diagnosis and long-term management, and most patients can expect favorable outcomes with appropriate care. The collective data underscore the importance of preventive strategies and highlight ONI as a largely avoidable, yet treatable, complication of modern pelvic surgery.
